# The challenging management of borderline ovarian tumors (BOTs) in women of childbearing age

**DOI:** 10.3389/fsurg.2022.973034

**Published:** 2022-08-23

**Authors:** Luigi Della Corte, Antonio Mercorio, Paolo Serafino, Francesco Viciglione, Mario Palumbo, Maria Chiara De Angelis, Maria Borgo, Cira Buonfantino, Marina Tesorone, Giuseppe Bifulco, Pierluigi Giampaolino

**Affiliations:** ^1^ Department of Neuroscience, Reproductive Sciences and Dentistry, School of Medicine, University of Naples Federico II, Naples, Italy; ^2^ Department of Public Health, University of Naples Federico II, Naples, Italy; ^3^ Department of Child and Adolescent Health, U.O.C Protection of Women's- ASL Napoli 1, Naples, Italy

**Keywords:** bordeline ovarian tumor, conservative surgery, ovary, fertility, recurrence, survival

## Abstract

Borderline ovarian tumors (BOTs) account for approximately 15% of all epithelial ovarian cancers. In 80% of cases the diagnosis of BOTs is done at stage I and more than a third of BOTs occurs in women younger than 40 years of age wishing to preserve their childbearing potential; the issue of conservative surgical management (fertility-sparing treatment) is thus becoming of paramount importance. At early stages, the modalities of conservative treatment could range from mono-lateral cystectomy to bilateral salpingo-oophorectomy. Although cystectomy is the preferred method to promote fertility it can lead to an elevated risk of recurrence; therefore, an appropriate counseling about the risk of relapse is mandatory before opting for this treatment. Nevertheless, relapses are often benign and can be treated by repeated conservative surgery. Besides the stage of the disease, histological subtype is another essential factor when considering the proper procedure: as most mucinous BOTs (mBOTs) are more commonly unilateral, the risk of an invasive recurrence seems to be higher, compared to serous histotype, therefore unilateral salpingo-oophorectomy is recommended. In the appraisal of current literature, this review aims to gain better insight on the current recommendations to identify the right balance between an accurate staging and an optimal fertility outcome.

## Introduction

Borderline ovarian tumors (BOTs) are a heterogeneous group of neoplasms with recognized potential malignancy, histologically defined by epithelial proliferation and nuclear atypia without recognizable destructive stromal invasion. Similar to carcinoma they can spread to the peritoneum and eventually to lymph nodes and in some patients can recur (1).

Peritoneal spread is present in 10% of BOTs and is divided into non-invasive (nearly 85% of implants) or invasive (2); the mortality rate for patients with non-invasive and invasive implants is 4.7% and 34% respectively (3).

BOTs are staged according to the FIGO staging system used for ovarian carcinoma (4). Most of the BOTs have a low potential for malignancy and are confined to the ovaries at presentation: unlike ovarian carcinoma in nearly 80% of cases the diagnosis is done at stage I with <1% of women diagnosed at stage IV (5).

Borderline ovarian tumors survival is 95% at 5 years, 90% at 10 years for women with FIGO stage I-III and nearly 77% at stage IV (6).

The vast majority of BOTs have serous or mucinous histotypes; about two-thirds are serous BOT (Table 1). Other rare types (<5%) are clear cell, endometrioid and Brenner tumors. A description of the natural behavior of the different histotypes is essential for the selection of the most appropriate surgical strategy.

**Table 1:** Main characteristics of the two most common BOTs histotypes.

	Serous borderline ovarian tumor	Mucinous borderline ovarian tumor
Prevalence	Almost 2/3 of all BOTs	More than 1/3 of all BOTs
Overall survival rate	Around 97%	Around 94%
Localization	Often bilateral	Nearly always unilateral
Peritoneal spread	30% of cases	Less frequent
Dimension (average diameter)	10 cm	20 cm
Relapse	More frequent	Less frequent
Type of recurrence	Generally non invasive (except for micropapillary patterns)	More often invasive
Predictivity of frozen section	Higher	Lower

**Figure 1:**
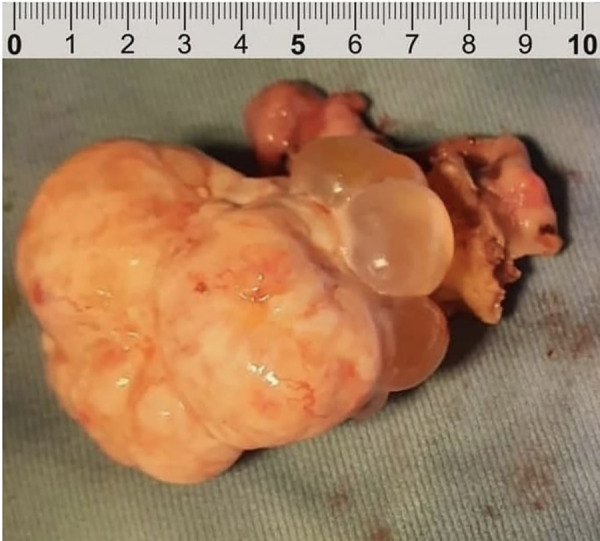
Ovarian serous borderline tumor.

Serous borderline ovarian tumors (sBOTs) are bilateral in 15%–25% of cases and noninvasive peritoneal spread is present in 15%/40% of cases (7) (Figure 1). The risk of invasive peritoneal spread is very low in early-stage serous tumors; only in a small percentage of cases do the implants infiltrate the underlying subperitoneal tissue and should thus be considered, according to the 2014 WHO (World Health Organization), as low-grade serous carcinoma (8).

The micropapillary/cribriform pattern is a variant of the common sBOT.

This lesion is defined by distinct morphological criteria and is more likely associated with a higher rate of bilateral ovarian involvement, recurrence, and invasive peritoneal implants compared with serous lesions without micropapillary patterns (9).

Mucinous borderline ovarian tumors (mBOTs) represent the second most common histologic subtype accounting for 30%–50% of all borderline ovarian tumors. Mucinous borderline tumors are nearly always unilateral and tend to be larger than sBOTs (average diameter of 20 cm) (Table 1) (10). Patients with mBOTs relapse less frequently than patients with serous disease, but when an extraovarian relapse occurs, the risk of an invasive recurrence and possible death seems to be higher (11).

## Methods

An electronic database search (Pubmed, Medline and Embase) was performed up to April 2022.

A search algorithm was developed incorporating the following medical terms “borderline ovarian tumors”, “low malignant potential”, “conservative surgery”, “fertility-sparing surgery”, “laparoscopy”, “invasive implants”, “micropapillary patterns”, “recurrence”.

All pertinent articles evaluating the diagnostic and therapeutic approaches centered on fertility-sparing treatment of borderline ovarian tumors have been included in this review.

All original studies, meta-analyses, systematic reviews and case reports published in English were considered. The reference lists were systematically reviewed to identify other studies for potential inclusion in this narrative review.

## Surgical approach

Fertility sparing surgery (FSS) has been defined as the preservation of the uterus and ovarian tissue in one or both adnexa (12). More than a third of BOTs affect women of reproductive age who wish to preserve their fertility potential.

In the management of early-stage BOTs, FSS is the mainstay of treatment, as an alternative to radical surgery (13).

In the advanced stages of the disease, the oncological safety of conservative treatment has still to be clarified (14). As a rule, patients with advanced-stage BOT should be considered not amenable to conservative surgical therapy in the presence of invasive implants or noninvasive implants not completely resectable (15–17).

Great attention has been recently focused on uterine preservation in the management of patients where preservation of healthy ovarian tissue is not feasible (18).

Indeed, ovarian tissue cryopreservation at the time of surgery, oocyte freezing, oocyte donation or a transfer of frozen embryos obtained before the surgical procedure are proposed to permit fertility (12, 19, 20).

FSS does not seem to affect the overall survival (21) however conservative treatment has been found to increase the relapse rate and therefore it is necessary to give full information to the patients about this risk.

Although the definitive diagnosis requires pathological evaluation after surgical excision, however preoperative knowledge of the specific ultrasonographic and macroscopic MRI features to differentiate BOTs subtypes can be extremely helpful to promote optimal patient management (22, 23).

The Frozen section (FS) plays an additional important role in determining the appropriate surgical management however, the surgeon should be aware of the well-known limitations of FS. The diagnostic accuracy rate for FS remains high for benign and malignant ovarian tumors but is relatively low for BOTs.

Frozen samples tend to under diagnose BOT as benign tumors in 25%–30% of cases, and improperly identify BOTs as carcinoma in 20%–30% of cases (24, 25). More caution in the use of FS in BOTs is needed, especially in cases of bulky tumors, where the intraoperative histology may lead to misdiagnosis of some features (e.g., microinvasion, papillary variant,intraepithelial carcinoma, stromal microinvasion) (26–28).

Although surgical staging does not have a significant impact on survival rate (29), nevertheless an initial complete staging appears to significantly reduce recurrence among BOTs patients (30). A complete exploration of the abdominal-pelvic peritoneal cavity, peritoneal washing, multiple peritoneal biopsies, infracolic omentectomy and complete resection of the implants for staging purposes are recommended (31).

A primary task for the surgeon is the complete removal of all peritoneal implants for both staging and therapeutic purposes with wide resection of surrounding tissue to allow the pathologist to discriminate non-invasive from invasive implants (32).

For the aforementioned reasons, surgical restaging should be considered in patients at higher risk of malignancy (mBOT, micropapillary variant, etc.) who underwent incomplete visual exploration of the abdominal pelvic peritoneum at the first surgery (33).

Lymph nodes involvement has a low prognostic value (34). In a retrospective analysis by Matsuo et al., no difference was found in survival rates in patients undergoing lymphadenectomy (35).

Lymphadenectomy is usually suggested only for cases with enlarged lymph nodes or invasive tumors detected on frozen examination (36, 37).

Obtaining a biopsy for the histopathologic evaluation from a normal appearing contra-lateral ovary is not helpful to reduce the risk of recurrence; an accurate preoperative ultrasonographic examination and a careful intraoperative macroscopic inspection is considered adequate for this purpose (38).

The use of minimally invasive or traditional open surgery has been evaluated in the literature: whatever the approach used, rupture of an intact tumor during its dissection/removal could alter the FIGO staging and affect the risk of recurrence (36).

In the last years, the use of minimally invasive procedure has increased dramatically, because of their reduced postoperative complications, blood loss, shorter postoperative recovery and cosmetic results. However, the decision on the surgical approach for BOT patients should be based on preoperative diagnostic features, epidemiological aspect and the surgeon's skill.

Published data report an increased intraoperative tumor rupture during laparoscopy cystectomies and identifies tumor volume as the main predictor factor.

Indeed, in a retrospective study of 105 patients, the tumor rupture was significantly more frequent during laparoscopy compared to laparotomy (29.5% vs. 13.1%, *p* = 0.038) (39). The conversion laparotomy rates is reporting of approximately 30% for BOT patients (40).

In another retrospective analysis, adnexa larger than 10 cm in maximum diameter were associated with a 4-fold risk of surgical spillage with laparoscopic approach (54.5% vs. 12.1%) compared to open surgery (37).

The laparoscopy compared to laparotomy has not shown a negative impact in terms of the recurrence rate, the survival and the feasibility of surgical management of BOTs (41). If surgery without risk of tumor rupture is possible, then the laparoscopic approach could be considered feasible, safe and recommended over laparotomy (40).

Robotic surgery is a feasible alternative in managing ovarian cancer as long as there is careful consideration given to patient selection (42). Robotic surgery is considered an option for the treatment borderline ovarian tumors, however the haptic feedback allowing to measure tissue traction and avoid cyst rupture is present only in some robotic platforms (43). Prospective randomized studies are needed to determine the relevance of robotic surgery in this context.

Another ultra minimally invasive approaches is the mini-laparoscopy that represents a great challenge for adnexal disease. Gueli Alletti et al. (44) have described a successful case of conservative staging surgery through the use of 2.4 mm needleoscopic instruments concluding that this could prove a beneficial tool in borderline disease.

## Adnexal surgery: What is best?

There is a lack of clear international guidelines on the optimal FSS procedure. FSS in stage I include unilateral/bilateral cystectomy, unilateral salpingo-oophorectomy and unilateral salpingo-oophorectomy plus contra-lateral cystectomy.

The impact of the histological subtype and the presence of factors associated with poor prognosis (microinvasion, micropapillary pattern, peritoneal implants) on FSS approach is relevant (45). As most mBOTs are at high risk of invasive recurrence and co-existence with invasive cancer areas is possible, unilateral salpingo-oophorectomy is considered the preferred surgical treatment in these cases (46); cystectomy is admissible only in presence of bilateral mBOT or when controlateral cystectomy is the only method to preserve fertility in patients with previous salpingo-oophorectomy (47).

Concerning sBOTs, often bilateral and characterized by a relatively benign behavior compared to mBOTs, the theoretically reproductive advantage of cystectomy as opposed to unilateral salpingo-oophorectomy is still waiting for a definitive conclusion.

The reproductive outcome seems not different between unilateral oophorectomy and cystectomy (48) on the other hand, but the available literature has raised concerns about a higher recurrence rate after cystectomy (49).

In a French multicenter study, including 313 patients with stage I BOTs, the recurrence rates after cystectomy, unilateral salpingo-oophorectomy or bilateral salpingo-oophorectomy have been found as 30.3%, 11% and 1.7%, respectively (50).

These results have been confirmed in a recent systematic review reporting the rate of recurrence correlated with the type of conservative surgery with a higher rate after cystectomy (41).

As opposite, Palomba et al. report that the use of bilateral cystectomy compared with a unilateral salpingo-oophorectomy and a contralateral cystectomy (in patients with bilateral BOTs, mainly in serous subtype) increases the fertility rate without increasing the recurrence rate (51). Vasconcelos et al. confirmed these results in a meta-analysis showing that, in case of bilateral serous BOT, unilateral salpingo-oophorectomy + contra-lateral cystectomy did not obtain any advantage compared to bilateral cystectomy in terms of recurrence (26.1% vs. 25.6%) (52). Standing this unresolved dispute, it is wise to concludethat whenever cystectomy is the selected procedure then appropriate counseling is recommended about a possible higher risk of local and peritoneal recurrence compared with salpingo-oophorectomy.

Obviously, cystectomy or unilateral salpingectomy + contra-lateral cystectomy remains the only fertility-sparing option in case of bilateral sBOT and in rare cases of previous surgical salpingo-oophorectomy (53).

## Disease recurrence

Conservative management has a significant impact on the development of BOTs recurrence if compared with radical surgery (5%–34% vs. 3.2%–7%) (52, 53).

The overall risk of recurrence varies between 2% and 24% and the risk of invasive recurrence ranges from 0.5% to 3.8%. Recurrences are seen in the remnant ovary after cystectomy, or in contralateral ovary or as extraovarian peritoneal and omental implants (54).

Complete surgical eradication of ovarian tumors and peritoneal implants even if not visible macroscopically is the prerequisite in minimizing the risk of disease relapse; pre- and intra-operative ultrasounds are of invaluable help to accomplish this goal (55).

Twenty-five percent of recurrences are diagnosed after 5 years (56); however, the recurrence rate is time-dependent and relapses may occur 15 years after surgery.

During the first two post-operative years, recurrences seem to be more frequent; a close follow-up is needed for this period through a systematic clinical examination including transvaginal ultrasonography and serum markers (57); unfortunately, only 40% of women with stage I BOTs have elevated levels of Ca125 and can benefit of this diagnostic measure (58, 59).

Most of sBOTs recurrences are in greater part borderline tumors, easily treated by repeated conservative surgery in patients desiring to preserve fertility (60).

Concerning mBOTs, if the risk of relapse is significantly lower compared with sBOTs, on the other side the risk of an invasive recurrence is higher (61).

Along with the histotype, some further clinico-pathological factors, although not unanimously, are considered helpful to identify patients more prone to invasive recurrence (60, 62).

Early-stage according to FIGO classification is a well-known independent risk factor for recurrence (63, 64); indeed, the rate of extraovarian recurrence has been demonstrated higher in stage IC3 and grade 3 tumors and consequently such aspects should be recognized as limits of conservative management for oncology safety (65).

Other factors such as micropapillary pattern and stromal microinvasion are histological aspects featuring a high-risk group likely to develop an invasive recurrence (57, 66).

Serous borderline ovarian tumors with micropapillary patterns seem to be more commonly associated with advanced stage, bilateral ovarian involvement, and invasive recurrence than the typical sBOTs (67, 68).

Notably, serous BOT displaying a micropapillary pattern without implants (stage I) or with non-invasive implants (stage II and III) could have the same prognosis as serous BOT without a micropapillary pattern (69).

On this basis, in case of micropapillary serous BOT without invasive implants it could be reasonable to propose a conservative approach only if combined with a careful and long follow-up (70); radical surgery to avoid any recurrence should be considered in those patients who completed their reproductive plans after conservative surgery or in cases without follow-up opportunities (71).

Data from literature identifies, stromal microinvasion, defined as a lesion that invades the stroma to a depth of 5 mm or less as a predictor of relapses: In a case series evaluating follow up data of 171 borderline mucinous tumors,microinvasive pattern was associated with an higher reuccrence rate (*p *= 0.013). In particular, in the group without microinvasion the rate of recurrence was 1.7% (2 of 116 cases), whereas in the group with microinvasion 14.3% (4 of 28 cases). No significant association was reported between clinicopathologic variables of these tumor and recurrence (72).

Higher rate of recurrence were also reported in a a retrospective study conducted on 902 patients with BOTs. Patients with microinvasive BOT had a significantly higher rate of recurrence than patients without microinvasive BOT (17.4 vs. 7.8%, OR 3.55, 95% CI 1.091–11.59, *p* = 0.03) . In particular stromal microinvasion was found as a prognostic factor for significantly shorter disease free survival (26.7 vs. 11.9 months, *p* = 0.031) (73).

In addition, data from 209 patients confirm that microinvasive BOTs recurred earlier with respect to noninvasive BOTs, with the median time to recurrence of 10.5 months for the first one and 17 months for the latter .For these patients unilateral salpingo-oophorectomy instead of cystectomy seems to not prevent relapses in microinvasive BOTs, that were recorded in 27% of the patients (74). However, in the studies, the overall survival seems to not differ significantly from BOTs without microinvasion.

Regrettably, microinvasion is associated with high frequency with the micropapillary variant in the serous BOTs and this is a potential confounding factor to identify its exact role in the genesis of recurrence (75–77). Due to these uncertainties, also for these patients, fertility-sparing surgery may be a reasonable option in young patients with BOTs, only if an accurate and strict follow-up is possible (78). Frequency and types of exams to perform in the follow-up surveillance are not established (79).

## Fertility outcomes after fertility-sparing surgery

Studies provide inconclusive findings about the impact of fertility-sparing treatments for BOTs on ovarian function (80) and an unanswered question remain whether pregnancy outcome is determined by the type of conservative approach (unilateral salpingo-oophorectomy/ovarian cystectomy). It is clear that ovarian surgery, especially after a second attempt, may reduce healthy ovarian parenchyma, increasing the risk of infertility. Moreover, the occurrence of postoperative adhesions might interfere with fallopian tube function (80–83).

However, after fertility-sparing surgery, pregnancy outcomes are encouraging and most pregnancies are achieved spontaneously as early as 3 months after surgery (84).

To avoid pregnancies complicated by recurrent disease, many physicians recommend delaying pregnancy until a sufficient follow-up period after initial surgical treatment (85, 86).

Little is known about the incidence and management of BOTs during pregnancy; however, expectant management could be a safe option in case of recurrences in pregnancy (87).

There is no specific data on the management of infertility following conservative treatment of BOTs and it is unclear if there is a potential impact of the use of fertility drugs on the recurrence rate of the disease (88).

Further data are needed on this topic considering that induction of ovulation and *in vitro* fertilization may be required in order to enhance the chance of conceiving.

## Borderline ovarian tumors during pregnancy

Little is known about incidence and management of BOTs during pregnancy. In reported literature, Borderline tumors diagnosed in pregnancy contain features concerning for aggressive behavior if compared to those diagnosed in non pregnant patients. A higher incidence of advanced stage at presentation as well as an higher higher percentage of mucinous BOT with intraepithelial carcinoma and microinvasion, and serous BOT with micropapillary component have been reported (89).

Unfortunately, concerning the management of BOT during pregnancy,only limited data, based mostly on case reports, are present in the literature.

As such, the standardization of the management strategy during pregnancy is difficult and, at the moment, it is based on the gold standard treatment of non-pregnant women.

An attitude of close surveillance should be adopted to exclude sign of malignant transformation (rapid enlargement of tumor, abnormal vascularization, presence of solid tissue) (90).

It is advisable that pregnancy and delivery are carried out in a tertiary center specialized in gynecology oncology. Technical difficulties of performing a complete staging for these patients at the initial surgery could necessitate a post partum completion staging or a debulking procedure and eventually an adjuvant chemotherapy (91).

## Conclusion

Fertility-sparing surgery is a well-established strategy available for patients with BOTs who desire to preserve fertility. This procedure is characterized by an excellent reproductive outcome and long-term survival.

Invasive recurrences remain one of the most important parameters of the safety of FSS. Unfortunately, the paucity of available data does not permit a definite identification of the prognostic factors of recurrence and makes the extent of conservative surgery as well as the modalities of a careful and effective post-operative follow-up still matter of debate.

Additional well-designed prospective studies, with larger samples, are needed to clarify these unresolved issues.
